# Fuzzy Comprehensive Evaluation-Based Disaster Risk Assessment of Desertification in Horqin Sand Land, China

**DOI:** 10.3390/ijerph120201703

**Published:** 2015-02-03

**Authors:** Yongfang Wang, Jiquan Zhang, Enliang Guo, Zhongyi Sun

**Affiliations:** School of Environment, Northeast Normal University, Changchun 130117, China; E-Mails: wangyf105@nenu.edu.cn (Y.W.); guoel675@nenu.edu.cn (E.G.); sunzy025@nenu.edu.cn (Z.S.)

**Keywords:** Horqin Sand Land, desertification, natural disaster risk, risk assessment

## Abstract

Desertification is a typical disaster risk event in which human settlements and living environments are destroyed. Desertification Disaster Risk Assessment can control and prevent the occurrence and development of desertification disasters and reduce their adverse influence on human society. This study presents the methodology and procedure for risk assessment and zoning of desertification disasters in Horqin Sand Land. Based on natural disaster risk theory and the desertification disaster formation mechanism, the Desertification Disaster Risk Index (DDRI) combined hazard, exposure, vulnerability and restorability factors and was developed mainly by using multi-source data and the fuzzy comprehensive evaluation method. The results showed that high risk and middle risk areas account for 28% and 23% of the study area, respectively. They are distributed with an “S” type in the study area. Low risk and very low risk areas account for 21% and 10% of the study area, respectively. They are distributed in the west-central and southwestern parts. Very high risk areas account for 18% of the study area and are distributed in the northeastern parts. The results can be used to know the desertification disaster risk level. It has important theoretical and practical significance to prevention and control of desertification in Horqin Sand Land and even in Northern China.

## 1. Introduction

China is one of the most serious desertification hazard countries in the world and Northern China is a key area of desertification research, prevention and control [1]. Although desertification research and control has been carried out for half a century, there is a sign that the situation is improving in parts, but the overall area is getting worse [2]. Desertification results in land degradation, lower biological production, loss of available land resources and deterioration of the ecological environment. It not only disturbs and affects the human survival and normal economic activities, but also creates huge losses in terms of lives and property. According to statistics, the economic losses caused by desertification disasters costs the country more than 54.1 billion Yuan a year [3]. Visible, in-depth studies on the desertification disaster problem are imperative.

Horqin Sand Land is located in the transition zone between northeast plain and Inner Mongolia plateau, it is a semi-humid and semi-arid region. It is one of the most serious desertification regions in arid and semi-arid areas in Northern China. Desertified land (the region affected by the desertification) in Horqin Sand Land measure 5 × 10^4^ km^2^, which accounts for 42% of the Sand Land. Compared with the 1950s, the ratio of desertified area has increased by 20%. The expansion of desertified land has doubled after half a century [4]. Such a wide range of land desertification has seriously hindered the development of the local society and economies, deepening the extent to which the people live in poverty. Therefore, desertification prevention and control has become one of the urgent problems to be solved in local and related areas.

Desertification research work has been carried out for many years all over the world and desertification assessment is an important related research field. From the existing research results, we have found that many of them are about monitoring and assessment of the desertification degree [5,6,7,8,9,10,11]. However, these are only about judgment or assessment of natural strength of desertification processes and desertification land types, with no thought for the disaster-causing ability of desertification and the interaction between the social and economic development and the losses, caused by disasters, on human society, which cannot reflect the essence of disaster. Disaster risk assessment is a quantitative analysis and assessment of the possibility of risk areas suffering from disasters of varying intensity, and their possible consequences. It not only considers the disaster itself, but also emphasizes the dangers of the disaster. As such, identification and assessment of risk factors of the desertification disaster can combat the occurrence and development of the desertification. It has more practical significance for desertification prevention and control.

Currently, only a few reports on disaster risk assessment of desertification exist. Based on a comprehensive environmental assessment model, Mouat selected five kinds of desertification risk assessment indicators (potential wind erosion, drought indicator, vegetation indicator, weed invader species and forage grass pressure indicator), which are used to assess the desertification risk in Colorado [12]. Sun used statistical modeling techniques to develop a desertification risk indicator (RI) for Minqin County, Gansu Province, China [13]. Costantini assessed desertification disaster risk in Italy by using a soil aridity indicator [14]. From the perspective of climate change and human disturbance, desertification risk was monitored by remote sensing and GIS technique in India [15]. Le considered many human activity factors (population, poverty, weak management capacity, lack of awareness, *etc.*) which were used to assess the desertification risk in Binh Thuan Province (Vietnam) by using the Leopold matrix [16]. Vanmaercke discussed how sediment yield can be used as a desertification risk indicator at the small regional scale [17]. A desertification prediction model was built based on the GIS technique and cellular automata by Chen [18]. Li carried out long-term monitoring and early warning research for Xinjiang, China [19]. Farajzadeh evaluated the desertification hazard for the Iyzad Khast plain, Iran, by using the MEDALUS model and GIS [20]. Gad mapped the environmental sensitivity areas for the desertification of Egyptian territory by using remote sensing and GIS [21]. Ladisa used a GIS-based approach and assessed the desertification risk in Apulia region of Southeastern Italy [22].

From the research content, the above studies are about the assessment of the possibility of the occurrence and development of desertification without considering the possibility of losses to human society; most studies used single indicator to characterize the desertification risk, few studies were characterized with a comprehensive indicator and the data source was single; obviously, there were not many researches based on the formation mechanism of the desertification risk. From the trend of the research, it is still necessary to carry out desertification risk assessment from its process and mechanism. In this way, the impact factors of the desertification can be more clearly seen, and their roles, which can then be used to serve the desertification prevention practice.

The main objectives of this study are to (1) build the concept framework of desertification disaster risk based on the theory of natural disaster risk and desertification disaster formation mechanism, (2) combined with the natural conditions and socio-economic situation of Horqin Sand Land, build an indicator system of desertification disaster risk assessment and Desertification Disaster Risk Index (DDRI) by using multi-source data (meteorological data, social and economic statistical data, remote sensing data and soil experimental data), (3) assess the desertification disaster risk level in Horqin Sand Land by using the entropy combination weighted method, fuzzy comprehensive evaluation method, optimal segmentation method and gridding GIS technique. The assessment results can help to recognize the desertification disaster risk level, and provide important references for the rational exploitation and utilization of lands. It also has important theoretical and practical significance for combating desertification in Horqin Sand Land even in the Northern China.

## 2. General Description of the Study Area

**Figure 1:**
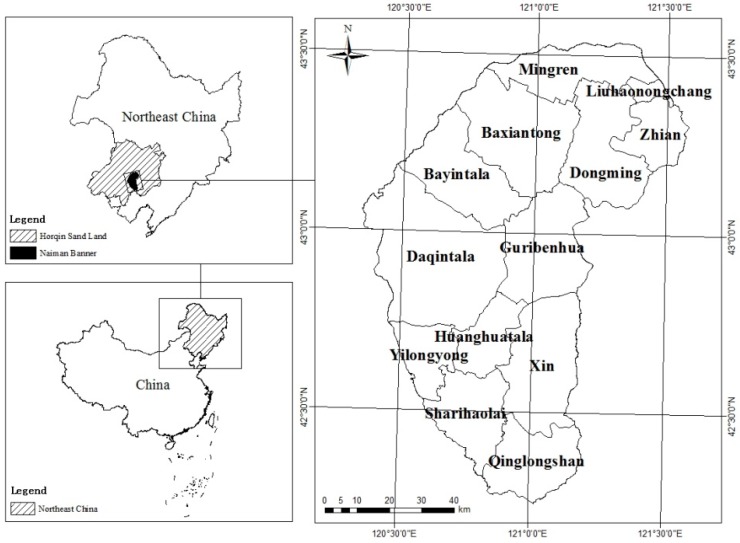
Location of the study area in Horqin Sand Land and Northeast China.

Naiman Banner is located in southern part of Tong Liao City, Inner Mongolia, China, which belongs to the south area of Horqin Sand Land. Geographical coordinates are from 120°19ʹ40ʺ E–121°35ʹ40ʺ E, 42°14ʹ40ʺ N–43°32ʹ20ʺ N, the total area is 8120 km^2^ (Figure 1). Naiman Banner has a continental semi-arid climate; altitude is 261–455 m, an average annual precipitation is 343.3–451.4 mm and mean evaporation is 1972.6–2081.8 mm. The area prevailed southwest wind in spring and northwest winds in winter, annual average wind speed is 3.5–4.1 m/s, and there are 20–60 windy days. Zonal soil is chestnut soil, but under the effect of wind erosion, many parts have degraded into Aeolian sandy soil (according to the Chinese soil classification system) [23]. Natural vegetation is woodland steppe, but is changed under the effects of human influence, especially in the north. Natural vegetation has been replaced by shrubs or subshrub. The area of the grassland is 2978.19 km^2^, which accounts for 36.8% of the total area, and is the largest land use pattern in the study area. The area of farmland is 2424.87 km^2^, which accounts for 29.9% of the total area, and is the second largest land use pattern in the study area. The area of the woodland is 801.97 km^2^, which accounts for 9.9% of the total area. The area of the residents mining land is 250.68 km^2^, which accounts for 3.1% of the total area. The area of the unused land area is 1578.54 km^2^, which accounts for 19.5% of the total area. The area of the water is 66.83 km^2^, which accounts for 0.8% of the total area. The economic development level of Naiman Banner is relatively backward.

Over the past century, massive vegetation has been damaged and land desertification has been serious for the increase in population and unrestrained over-reclamation and overgrazing. According to the analysis, in the late 1950s, the area of desertification land in Naiman Banner was 2399 km^2^, accounting for 29.1% of the total land area. In the late 1970s, the area of land affected by desertification in Naiman Banner was 5657 km^2^, accounting for 69.7% of the total land area. The area of desertification land has decreased with desertification combating as the following years. By 2009, the area of desertification land in Naiman Banner was 4155.41 km^2^, accounting for 51.29% of the total land area [24]. We can clearly see that although the area of desertification land has decreased, it still accounts for more than half of the total area. Naiman Banner is one of the most typical desertification regions in Horqin Sand Land even in Northern China.

## 3. Study Approach and Data Treatments

### 3.1. Study Approach

#### 3.1.1. Entropy Combination Weighted Method

The Analytic Hierarchy Process (AHP) is a convenient and effective method that implements qualitative and quantitative analysis for indicators and compares the indicators one to one. It not only can be conducted continuously, but also can be readily improved. The indictors are subdivided into different membership levels according to the tightness of its class. They are quantified on the basis of one indicator and different components of each indicator reflect the influence degree on the research object [25]. The AHP has a certain subjectivity. The entropy weight method can objectively reflect the weight of each indicator. Its basic principle is that the smaller the information entropy (which indicates the ratio of the amount of information and the information value, the lower the entropy value is, the bigger the information values, indicating the relative importance of the indicator ) of an indicator is, the greater the degrees of the variation of the indicator values. The greater the amount of information it provided, the larger the role it played in comprehensive evaluation and the greater the weight it has [26]. Using the minimum relative entropy principle, entropy combination weighted method combined the AHP and entropy weight method to reduce the impact of subjective and objective influence. Entropy combination weighted is calculated by the Formula (1).

(1) $$W_{j}=\sqrt{\left(W_{1j}\times W_{2j}\right)}/\sum (\sqrt{W_{1j}\times W_{2j}})$$

where $W_{j}$ is the comprehensive weight of indicator j; $W_{1j}$ is the subjective weight of indicator j; $W_{2j}$ is the objective weight of indicator j.

#### 3.1.2. Optimal Segmentation Method

The optimal segmentation method can be used to classify the ordered sample or the sample can be transformed into an ordered sample [27]. Segmentation is carried on the condition that the data must be ranged in ascending order. The concrete methods include optimal two-segmentation, optimal three-segmentation and optimal M-segmentation.

#### 3.1.3. Gridding Geographic Information System

The scale determines the precision of the risk assessment work. Risk levels in different areas have a certain difference for the spatial heterogeneity and varying degrees of disturbance. The gridding GIS takes a grid as research object, analyzes attribute data such as natural, social and economic, in order to express the differences among the attributed data of each grid. A spatial database is constructed with statistical data within the administrative divisions unit, which is distributed in certain grids.

This not only facilitates the statistics of various data and natural application of the geographic features, but also improves the degree of compliance between calculated results and the actual situation. We gridded the study areas, made each risk indicator distribute within each grid, and carried out visual expression to the regional desertification risk assessment results [28].

#### 3.1.4. Fuzzy Comprehensive Evaluation Method

Fuzzy comprehensive evaluation is a kind of method that analyzes and evaluates the fuzzy system by the fuzzy transformation principle [29]. The method shows a unique superiority in dealing with complex system problems that could be described by the precise mathematical method. Fuzzy mathematics characterizes the degree of elements belonging to a set by using membership and expanding binary logic of Two-valued logic classic set (0, 1) to continuous-valued logic of Interval [0, 1] to provide an effective means for description and reaction of various fuzzy things and phenomena.

The main steps of the fuzzy comprehensive evaluation method are as follows.

(1) To determine the evaluation indicator set U. $U=\left\{u_{1},u_{2},\ldots ,u_{n}\right\}$, of which $u_{1},u_{2},\ldots ,u_{n}$ are the value of each evaluation indicator of evaluation object.

(2) According to the demand of evaluation decision, evaluation class will be divided into five grades (very high, high, middle, low and very low) by using the optimal segmentation method. Determine the evaluation grade set V and set up $V=\left\{v_{1},v_{2},\ldots ,v_{n}\right\}$ in which $v_{1},v_{2},\ldots ,v_{n}$ are the each evaluation grade.

(3) To determine the fuzzy relationship matrix R. Membership of evaluation grade of each indicator was determined on the basis of membership function. Relationship matrix of fuzzy sets R is as follows.

The fuzzy sets take the membership function as a bridge to convert uncertainty into certainty in form. This means quantifying the fuzziness and getting the fuzzy evaluation matrix. Membership function is cornerstone in the establishment of fuzzy set theory. Membership functions commonly used linear triangular distribution, trapezoidal distribution, normal distribution, parabolic distribution, *etc.* This article uses the trapezoidal distribution membership function; membership functions of increment type/decrement type indicators were given in the light of characteristics that the grade of indicator increases or decreases with the increasing of the indicator value.

**Figure 2:**
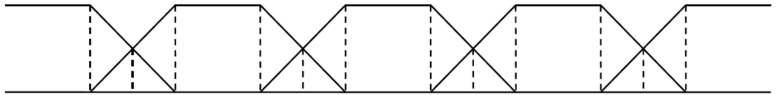
Diagram of decreasing type membership function.

For decreasing type indicators $x_{i}$, the membership is shown in Figure 2 and the membership function is calculated by the Formulas (2)–(6).

(2) $$v_{1}(x_{i})=\{\begin{array}{cc} 1 & {a_{i1}}^{+}\leq x_{i}\\ \frac{x_{i}-{a_{i1}}^{-}}{{a_{i1}}^{+}-{a_{i1}}^{-}} & {a_{i1}}^{-}<x_{i}<{a_{i1}}^{+}\\ 0 & else \end{array}$$

(3) $$v_{2}(x_{i})=\{\begin{array}{cc} \frac{{a_{i1}}^{+}-x_{i}}{{a_{i1}}^{+}-{a_{i1}}^{-}} & {a_{i1}}^{-}<x_{i}<{a_{i1}}^{+}\\ 1 & {a_{i2}}^{+}\leq x_{i}\leq {a_{i1}}^{-}\\ \frac{x_{i}-{a_{i2}}^{-}}{{a_{i2}}^{+}-{a_{i2}}^{-}} & {a_{i2}}^{-}<x_{i}<{a_{i2}}^{+}\\ 0 & else \end{array}$$

(4) $$v_{3}(x_{i})=\{\begin{array}{cc} \frac{{a_{i2}}^{+}-x_{i}}{{a_{i2}}^{+}-{a_{i2}}^{-}} & {a_{i2}}^{-}<x_{i}<{a_{i2}}^{+}\\ 1 & {a_{i3}}^{+}\leq x_{i}\leq {a_{i2}}^{-}\\ \frac{x_{i}-{a_{i3}}^{-}}{{a_{i3}}^{+}-{a_{i3}}^{-}} & {a_{i3}}^{-}<x_{i}<{a_{i3}}^{+}\\ 0 & else \end{array}$$

(5) $$v_{4}(x_{i})=\{\begin{array}{cc} \frac{{a_{i3}}^{+}-x_{i}}{{a_{i3}}^{+}-{a_{i3}}^{-}} & {a_{i3}}^{-}<x_{i}<{a_{i3}}^{+}\\ 1 & {a_{i4}}^{+}\leq x_{i}\leq {a_{i3}}^{-}\\ \frac{x_{i}-{a_{i4}}^{-}}{{a_{i4}}^{+}-{a_{i4}}^{-}} & {a_{i4}}^{-}<x_{i}<{a_{i4}}^{+}\\ 0 & else \end{array}$$

(6) $$v_{5}(x_{i})=\{\begin{array}{cc} 1 & x_{i}\leq {a_{i4}}^{-}\\ \frac{{a_{i4}}^{+}-x_{i}}{{a_{i4}}^{+}-{a_{i4}}^{-}} & {a_{i4}}^{-}<x_{i}<{a_{i4}}^{+}\\ 0 & else \end{array}$$

In the formulas (2)–(6),

$$\begin{array}{l} {a_{i1}}^{+}=a_{i1}+\frac{1}{4}\left|a_{i1}\right|,{a_{i1}}^{-}=\frac{3a_{i1}+a_{i2}}{4},{a_{i2}}^{+}=\frac{a_{i1}+3a_{i2}}{4},{a_{i2}}^{-}=\frac{3a_{i2}+a_{i3}}{4}\\ {a_{i3}}^{+}=\frac{a_{i2}+3a_{i3}}{4},{a_{i3}}^{-}=\frac{3a_{i3}+a_{i4}}{4},{a_{i4}}^{+}=\frac{a_{i3}+3a_{i4}}{4},{a_{i4}}^{-}=\frac{3a_{i4}}{4} \end{array}$$

**Figure 3:**
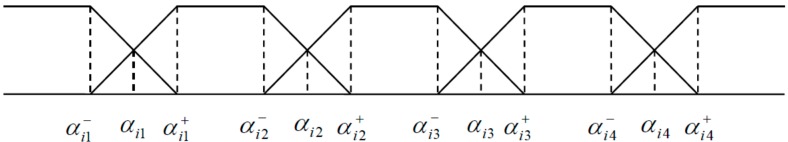
Diagram of increasing type membership function.

For increasing type indicators $x_{i}$, the membership is shown in Figure 3 and the membership function is calculated by Formulas (7)–(11).

(7) $$v_{1}(x_{i})=\{\begin{array}{cc} 1 & x_{i}\leq {a_{i1}}^{-}\\ \frac{{a_{i1}}^{+}-x_{i}}{{a_{i1}}^{+}-{a_{i1}}^{-}} & {a_{i1}}^{-}<x_{i}<{a_{i1}}^{+}\\ 0 & else \end{array}$$

(8) $$v_{2}(x_{i})=\{\begin{array}{cc} \frac{x_{i}-{a_{i1}}^{-}}{{a_{i1}}^{+}-{a_{i1}}^{-}} & {a_{i1}}^{-}<x_{i}<{a_{i1}}^{+}\\ 1 & {a_{i1}}^{+}\leq x_{i}\leq {a_{i2}}^{-}\\ \frac{{a_{i2}}^{+}-x_{i}}{{a_{i2}}^{+}-{a_{i2}}^{-}} & {a_{i2}}^{-}<x_{i}<{a_{i2}}^{+}\\ 0 & else \end{array}$$

(9) $$v_{3}(x_{i})=\{\begin{array}{cc} \frac{x_{i}-\;{a_{i2}}^{-}}{{a_{i2}}^{+}-\;{a_{i2}}^{-}} & {a_{i2}}^{-}<x_{i}<{a_{i2}}^{+}\\ 1 & {a_{i2}}^{+}\leq x_{i}\leq {a_{i3}}^{-}\\ \frac{{a_{i3}}^{+}-\;x_{i}}{{a_{i3}}^{+}-\;{a_{i3}}^{-}} & {a_{i3}}^{-}<x_{i}<{a_{i3}}^{+}\\ 0 & else \end{array}$$

(10) $$v_{4}(x_{i})=\{\begin{array}{cc} \frac{x_{i}-\;{a_{i3}}^{-}}{{a_{i3}}^{+}-{a_{i3}}^{-}} & {a_{i3}}^{-}<x_{i}<{a_{i3}}^{+}\\ 1 & {a_{i3}}^{+}\leq x_{i}\leq {a_{i4}}^{-}\\ \frac{{a_{i4}}^{+}-\;x_{i}}{{a_{i4}}^{+}-\;{a_{i4}}^{-}} & {a_{i4}}^{-}<x_{i}<{a_{i4}}^{+}\\ 0 & else \end{array}$$

(11) $$v_{5}(x_{i})=\{\begin{array}{cc} 1 & {a_{i4}}^{+}\leq x_{i}\\ \frac{x_{i}-\;{a_{i4}}^{-}}{{a_{i4}}^{+}-\;{a_{i4}}^{-}} & {a_{i4}}^{-}<x_{i}<{a_{i4}}^{+}\\ 0 & else \end{array}$$

In the formulas (7)–(11),

$$\begin{array}{l} {a_{i1}}^{-}=a_{i1}-\frac{1}{4}\left|a_{i1}\right|,{a_{i1}}^{+}=\frac{3a_{i1}+a_{i2}}{4},{a_{i2}}^{-}=\frac{a_{i1}+3a_{i2}}{4},{a_{i2}}^{+}=\frac{3a_{i2}+a_{i3}}{4}\\ {a_{i3}}^{-}=\frac{a_{i2}+3a_{i3}}{4},{a_{i3}}^{+}=\frac{3a_{i3}+a_{i4}}{4},{a_{i4}}^{-}=\frac{a_{i3}+3a_{i4}}{4},{a_{i4}}^{+}=a_{i4}+\frac{a_{i1}}{4} \end{array}$$

where $\alpha_{i1}$, $\alpha_{i2}$, $\alpha_{i3}$, $\alpha_{i4}$ are split points of the grade of the evaluation indicators. $v_{1}\left(x_{i}\right)$, $v_{2}\left(x_{i}\right)$, $v_{3}\left(x_{i}\right)$, $v_{4}\left(x_{i}\right)$, $v_{5}\left(x_{i}\right)$ are the membership values of indicator $x_{i}$ belongs to grade 1 to grade 5.

(4) To determine the weight vector of evaluation indicators. The weights of each indicator obtained by entropy combination weighted method are $a_{1},a_{2}\ldots ,a_{n}$, then $A=(a_{1},a_{2}\ldots ,a_{n})$

(5) Comprehensive evaluation. Set B=A×R, among which B is the comprehensive value of evaluation object, A is the weights of the evaluation indicators, R is the membership of the evaluation indicators for each evaluation grade. We can get the grade of the evaluation object by comparing B with the critical value of each evaluation grade.

### 3.2. Data Treatments

#### 3.2.1. Meteorological Data

Meteorological data used in this paper is from the China Meteorological Science Data Sharing Service and the Meteorological Bureau of Naiman Banner. We selected daily data from 17 meteorological stations, which are separately distributed in Inner Mongolia, Jilin Province and Liaoning Province, from 1970 to 2010.

#### 3.2.2. Socioeconomic Data

Parts of the socioeconomic data used in this paper are from the statistical yearbook of Naiman Banner from 1970 to 2010 and others are from the Forestry Bureau of Naiman Banner.

#### 3.2.3. Vegetation Coverage Index

We can get different types of vegetation index from by mean of combining two or more spectral channels. They reflect the evolution of vegetation information to some extent. So far, the normalized differential vegetation index (*NDVI*) has been widely used in more than 40 kinds of vegetation indices. It can effectively monitor vegetation conditions, estimate vegetation cover, leaf area index and other ecological parameters. It can also partially compensate for the influence of lighting conditions, the slope of the ground and the changes in satellite direction [30]. The value of the *NDVI* is calculated by the Formula (12).

(12) $$NDVI=\frac{NIR-R}{NIR+R}$$

where NIR and R present the reflectance of infrared band and red band. The range of the *NDVI* value is [−1, 1]. The greater the heights, groups, and leaf area index of the plants are, the higher the *NDVI* values are.

The vegetation coverage index (VCI) refers to the ratio of vertical vegetation projection area of vegetation canopy and the total area of the land. It is an important biophysical parameter to characterize the vegetation. The vegetation coverage index can be calculated through NDVI, it can be used to characterize the vegetation rate. The value of the *VCI* is calculated by Formula (13).

(13) $$VCI=\frac{\left(NDVI-NDVI_{\text{min}}\right)}{\left(NDVI_{\text{max}}-NDVI_{\text{min}}\right)}$$

where $NDVI_{\text{min}}$ is the minimum value of the *NDVI* and $NDVI_{\text{max}}$ is the maximum value of the *NDVI* in the study area.

Remote sensing data used in this paper is MODIS data with 16 days synthesis and 1 km resolution, which was released by The U.S. National Space Agency (NASA). We calculated the *NDVI* and the vegetation coverage index by using Band Math of ENVI 4.8 after atmospheric correction, projection conversion, mosaic and cutting of original remote sensing.

#### 3.2.4. Soil Experimental Data

We select soil texture (coarse sand, fine sand, silt and clay) as an indicator of soil physical properties and select soil nutrients (organic matter, total nitrogen, total phosphorus, total potassium, alkali-hydrolyzable nitrogen, available phosphorus and available potassium) as soil chemical properties. These indicators are derived from field sampling and soil experiments.

We randomly selected 10 samples for soil sampling in different types of lands (farmland and grassland) within 13 administrative regions in Naiman Banner. Of each soil sample, we collected three repeat samples and the weights of each soil sample was about 1kg. We then sent the soil samples for laboratory analysis.

Soil particle size analysis used the pipette method, organic matter used the potassium dichromate volumetric method, total nitrogen used the semi-micro's method, total phosphorus used the NaOH melt-molybdenum anti-calorimetric method, total potassium used the NaOH melt-flame photometry method, alkali-hydrolyzable nitrogen used the alkaline hydrolysis diffusion method, available phosphorus used the 0.5 mol·NaHCO_3_ method and available potassium used the NH_4_OA_C_ extraction flame photometric method [31].

We did spatial interpolation for sampling point data of soil physical and chemical properties of 11 indicators, and spatial distributions of each indicator were generated by using the Kriging interpolation method. We weighed the 11 indicators by using the entropy combination weighted method and superimposed them to get the spatial distribution map of the soil physical and chemical properties of the farmland and grassland.

## 4. Process of Establishing the Desertification Disaster Risk Assessment Model

### 4.1. Formation Principles and Conceptual Framework of Desertification Disaster Risk

Take sand activities as the main sign, desertification is a land degradation which caused by coordination between man-land relationships in arid, semi-arid and parts of sub-humid areas [32]. Desertification disaster risk refers to the possibility of the occurrence and development of desertification disasters and the possibility of loss caused by disaster on human society.

Based on the theory of natural disaster risk formation, desertification disaster risk is a result of the interaction of hazard (*H*), exposure (*E*), vulnerability (*V*) and restorability (*R*) [33]. The desertification disaster risk is obtained by Formula (14).

(14) Desertification disaster risk = H×E×V×R

Hazard is the variation degree of natural disasters and human factors which caused the desertification disaster. The greater the variation degree is, the greater the hazard and the disaster risk. Here, the natural factor refers to the fragile natural conditions caused desertification disaster. Less precipitation, high temperatures, high evaporation, windy weather can result in a desertification area with low vegetation coverage, which is more prone to desertification disaster risk; if the natural factors are the basis of the desertification happened, human factors are the promoting factors of desertification occurrence. The unreasonable way and extent to which land use has disturbed and destroyed native vegetation, creating favorable conditions for the desertification process, has made the desertification development speed increase exponentially. When human and natural forces are coupled, desertification develops dramatically.

Exposure (hazard bearing body) refers to the possibility of economic, social and natural environmental systems that may be threatened by hazard factors (natural and human factors), including agriculture, livestock, human, ecological environment, *etc.* The higher the value density exposure to desertification hazard factors, the greater the potential losses it would suffer. Desertification is a land degradation process, and if it happens or develops, it will lead to the decline of soil productivity, resulting in the decline of food production or forage yield. Thus, it affects agriculture and the development of animal husbandry, causing people to live in poverty. We can see that land systems (hazard bearing body) are directly exposed to desertification hazard factors and the population is indirectly exposed to the desertification hazard factors.

Vulnerability refers to the damage or loss degree that the hazard bearing body caused by potential risk factors in a given area. The lower the vulnerability the hazard bearing body has, the smaller the disaster losses and the disaster risks are, and vice versa. The vulnerability of the hazard bearing body relates to material composition, structure and disaster prevention efforts [34].

Restorability refers to variety measures and countermeasures for disaster prevention and mitigation. The greater the restorability is, the smaller the potential losses and the desertification disaster risks are. In the desertification area, desertification combating work has been carried out for many years. The main form is comprised of economic inputs (sand-control inputs), soil bioengineering construction, *etc.* In addition, population control and improving population quality (improving per capita level of education) are also effective to combat the occurrence and development of desertification.

**Figure 4:**
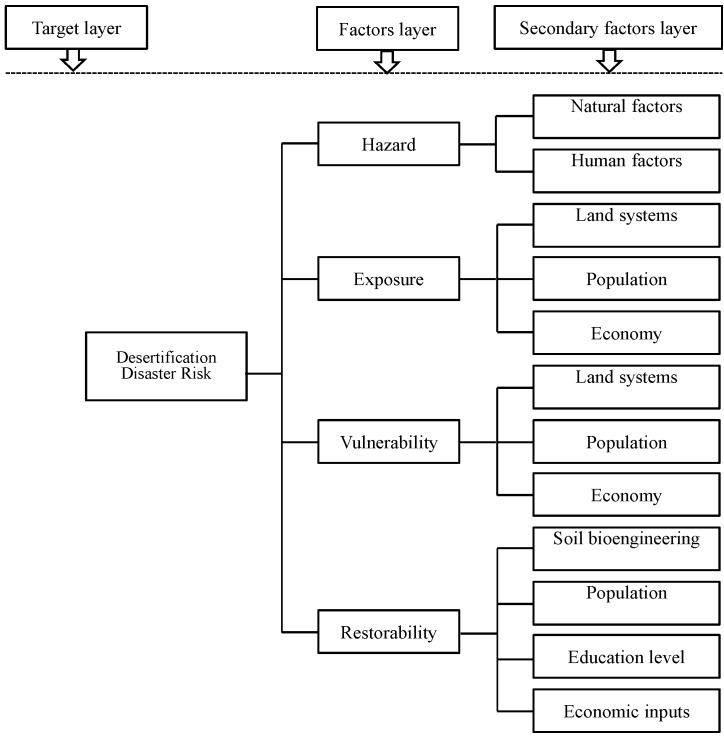
Concept framework of desertification disaster risk.

According to the forming principle of the desertification disaster risk, we have formulated the concept framework of desertification disaster risk, as shown in Figure 4.

### 4.2. Indicator System of the Desertification Disaster Risk Assessment

#### 4.2.1. Selection of Indicators

Based on the forming principle of desertification disaster risk and the natural, social and economic status in the study area, we selected 19 indicators in Table 1 to established desertification disaster risk assessment system. The indicator system can be divided into the target layer, factors layer, secondary factor layer and indicator layer.

**Table 1:** Desertification disaster risk assessment system.

Target Layer	Factors Layer	Secondary Factors layer	Indicators Layer
Desertification Disaster Risk Index of Naiman Banner	Hazard	Natural factors	precipitation
			evaporation
			sand driving wind days
			temperature
			vegetation coverage index
		Human factors	cultivation rate
			grazing capacity
	Exposure	Land systems	grassland area
			farmland area
		Population	population density
		Economy	economic density
	Vulnerability	Land systems	soil physical and chemical properties of grassland
			soil physical and chemical properties of farmland
		Population	ratio of agricultural population
		Economy	ratio of farming, forestry, husbandry and fishing outputs to GDP
	Restorability	Soil bioengineering measures	area of returning farmland to forests
		Population	population output
		Education level	number of students
		Economic inputs	ratio of sand-control inputs to GDP

We considered hazard factors from two aspects of natural factors and human factors. As already mentioned above, the area with fragile climatic conditions and low vegetation coverage are more easily prone to desertification disasters. (1) We selected the vegetation coverage index as the desertification disaster risk assessment indicator to demonstrate the vegetation coverage degree. The lower the vegetation coverage is, the greater the hazard and the risk of the desertification disaster. In the study area, poor weather conditions are the main natural hazard factors. High temperatures, less precipitation, and high evaporation will greatly increase the desertification risk. Wind is an important factor in sandstorm activities that may cause desertification. However, not all of the wind will be the work, desertification will be affected only when the wind speed reaches the critical sand driving wind speed. Therefore, we selected “sand driving wind days” as the desertification hazard indicator. (2) The human risk factor is mainly reflected in the irrational land use patterns by human. In the study area, the main land use patterns are grassland and farmland. On the one hand, the study area is a grazing area, but for the continuous expansion of farming lines, lots of grasslands are converted into farmland. Farmland is a land use type that does not exist in a natural state. Reclaiming grassland destroyed not only vegetation, but also the soil structure. Bare and loose surfaces are the most susceptible to wind erosion. On the other hand, overgrazing make the decrease of the grassland resources, the grass is deteriorating because of the excessive eating by animals, together with livestock trampling, the surface will be further exposed. Visibly, reclamation and grazing are the main human factors of the desertification risk in the area. We use the cultivation rate and grazing capacity to express them, respectively. The reclamation rate is a percentage of farmland to the total area, is an indicators that reflect the extent of land reclamation in a region. Grazing capacity represent the numbers of stocking livestock in per unit area. High grazing capacity not only destroyed the pasture, but also let them lose their function and exacerbated the occurrence and development of the desertification disaster.

In this paper, exposure includes land system exposure, population exposure and economic exposure. (1) Two main land use types in the study area are grassland and farmland, so we used grassland area and farmland area to express the exposure degree of the disaster bearing body. (2) Population exposure is represented by population density. Population density refers to the number of people per unit area. The greater the population density, the more people may withstand the desertification disaster. (3) Economic exposure is represented by economic density. Economic density refers to the GDP per unit area. The higher the economic density, the more economic losses may be caused by the desertification disaster.

Vulnerability is mainly related to the material composition, structure and state. (1) Desertification is a land degradation process whose occurrence and development will be firstly reflected in the changes to soil properties. Thus, we choose soil physical and chemical properties of the grassland and farmland to characterize the land system vulnerability. (2) Population vulnerability is represented by the ratio of agricultural population. Most of the agricultural population relies on the land to survive, so the greater the agricultural population, the bigger the possibility of the losses caused by the desertification disaster. (3) Economic vulnerability is represented by ratio of farming, forestry, husbandry and fishing outputs to GDP. Farming, forestry, husbandry and fishing all belong to the primary industry. High ratio of the primary industry outputs to the GDP represent the low level of the regional economic development. The study area is a farming-pastoral transitional zone dominated by farming and husbandry production activities. Weak economic conditions have increased the possibility of the losses caused by desertification disaster.

Restorability refers to variety measures and countermeasures for disaster prevention and mitigation. (1) Because of the ecologically fragile environment and widespread desertification land, the study area has implemented some soil bioengineering measures and invests in lots of sand-control funds. We select an area of farmland being returned to forest to demonstrate implementation of the ecological engineering situation. (2) We used ratio of sand-control inputs to the GDP to indicate the investment situation of sand control. (3) A high level of education not only can reduce the occurrence of irrational human activities but also can mitigate the pressure on land systems, thereby preventing the occurrence and development of desertification disasters. Education level is represented by numbers of students. Multiple numbers of students indicate that the possibility of rational utilization of land resources is large. (4) Controlling the number of the population is an effective method to reduce the pressure on land system. However, the data shows that there is no obvious growth in population. Therefore, we consider population outputs as restorability indicator. Population output of a region can reduce the pressure on the land system and can also bring additional income to local economies [1,2,28].

#### 4.2.2. Spatial distribution of the indicators

We did spatial interpolation to meteorological indicators and soil indicators by using kriging and the inverse distance weighting method and implemented in spatial analyst module of the ArcGIS10.0 software; we did spatial interpolation to partial social economic indicators by using the Cokriging method and implemented the geostatistical analysis module of the ArcGIS 10.0 software; some indicators are distributed by using grid distribution. The accuracy of the grid used in this paper is 1 km × 1 km.

#### 4.2.3. Weights and grading standards

We used the entropy combination weighted method to get the weight of the indicators and used the optimal segmentation method to divide the assessment levels of each indicator (Table 2).

#### 4.2.4. Establishing of Desertification Disaster Risk Assessment Model

Based on the forming principle of desertification disaster risk, we considered the four factors of desertification disaster risk and the indicator system, we established desertification disaster risk assessment model by using the entropy combination weighted method and the fuzzy comprehensive evaluation method. The DDRI is obtained by formula (15).

(15) $$DDRI=\left(H^{WH}\right)\left(E^{WE}\right)\left(V^{WV}\right){\left[1-R\right]}^{WR}$$

where DDRI is the desertification disaster risk index. The bigger the value, the greater the desertification disaster risk; Value of *H*, *E*, *V*, *R* express the Hazard factor index, the Exposure factor index, the Vulnerability factor index and the Restorability factor index respectively; *W_h_, W_e_, W_v_,* W*_r_* express the weights of hazard, exposure, vulnerability and restorability, respectively.

## 5. Results and Discussion

### 5.1. Assessment and zoning of Desertification Disaster Risk Four Factors

**Figure 5:**
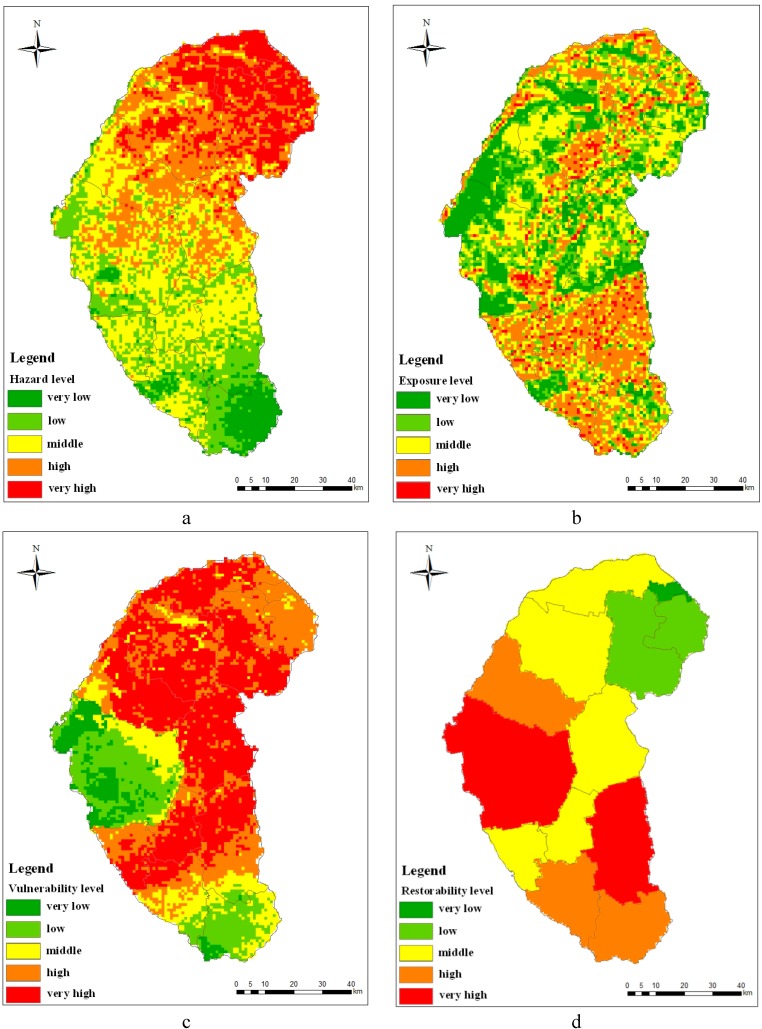
(**a**) Level zoning map of desertification disaster risk hazard. (**b**) Level zoning map of desertification disaster risk exposure. (**c**) Level zoning map of desertification disaster risk vulnerability. (**d**) Level zoning map of desertification disaster risk restorability.

We should consider the four factors which make up the desertification disaster risk. Based on the indicator system mentioned above, we got the level zoning map of the hazard, exposure, vulnerability and restorability, respectively, by using the fuzzy comprehensive evaluation method, as shown in Figure 5. In addition, we calculated the area ratio of the different types of factors by using the Spatial Analyst module of ArcGIS10.0 software.

A level zoning map of desertification disaster risk hazard in Naiman Banner is shown in Figure 5a. The area ratio of middle hazard is highest in the study area, and it accounts for 32% of the total area. High hazard area and very high hazard area comprise 25% and 16% of the total area, respectively. Low hazard area and very low hazard area comprise 20% and 7% of the total area, respectively. We can know that risks in most part of the study area are above the middle level. We can see from Figure 5a that the desertification disaster risk increased gradually from south to north on the whole. Relatively high risks in northern part of the study area are mainly due to less precipitation, windy weather and moderate vegetation coverage. Although the cultivation rate is high in the southern part, higher precipitation, less windy weather, higher vegetation coverage and lower grazing capacity lead to the low and very low hazard in this area. The central part of the study area is composed of middle hazard. This area has lower vegetation coverage, higher grazing capacity and moderate climate conditions compared with the northern part and the southern part.

A level zoning map of desertification disaster risk exposure in Naiman Banner is shown in Figure 5b. The same as the hazard, the area ratio of middle exposure is highest in the study area, and it accounts for 30% of the total area. The second is the high exposure area, accounting for 25%. Low exposure area and very low exposure comprise 22% and 19% of the total area, respectively. In addition, the very high exposure area comprised 4% of the study area. We can see from Figure 5b that the high exposure area is mainly distributed in the southern part, with parts distributed in the northern part of the study area. The very high exposure area is distributed sporadically with the high exposure area. In the south, the economic density and population density is relatively high and there are many farmlands, resulting in the high exposure. The low exposure, high exposure and middle exposure areas are mainly distributed in the southern part and some are distributed in more parts of the central and northern part of the study areas. There are many grassland resources in the north, so its desertification disaster risk exposure is relatively higher.

A level zoning map of desertification disaster risk vulnerability in Naiman Banner is shown in Figure 5c. The area ratio of very high vulnerability is highest in the study area, and it accounts for 42% of the total area. The second is the high vulnerability area, accounting for 26% of the study area. The middle vulnerability area, low vulnerability area and very high vulnerability area comprise 12%, 14% and 6% of the total area, respectively. We can see from Figure 5c that middle vulnerability area, low vulnerability area and very high vulnerability area are mainly distributed in parts of the south and west-central of the study area. This is mainly due to the good physical and chemical properties of farmlands and grasslands, and the low ratio of agricultural population here. A high vulnerability area is distributed with the “S” shape throughout the whole study area, accounting for almost half of the land area in the study area. This is mainly due to the high ratio of agricultural population, and the poor physical and chemical properties of soil. Furthermore, the area is mainly engaged in farming and husbandry. The higher outputs of the farming, forestry, husbandry and fishing (in the study area, the outputs are mainly from farming and husbandry), the greater the vulnerability of the economics of the area (because in economically developed areas, the proportion of farming and husbandry are relatively low, while the second and the third industries are relatively developed).

**Table 2:** Indicator system and its grading standard of the desertification disaster risk assessment.

Desertification Disaster Risk Four Factors	Indicators	Weights	Level 1	Level 2	Level 3	Level 4	Level 5
Hazard (0.4088)	precipitation	0.1025	(330.92, 348.33]	(348.33, 366.19]	(366.19, 388.51]	(388.51, 414.40]	(414.04, 444.76]
	evaporation	0.0321	(1813.9, 1846.4]	(1846.4, 1876.9]	(1876.9, 1927.3]	(1927.3, 1996.7]	(1996.7, 2081.8]
	sand driving wind days	0.0699	(53, 57]	(57, 60]	(60, 63]	(63, 67]	(67, 71]
	temperature	0.0338	(6.68, 6.80]	(6.80, 6.89]	(6.89, 6.98]	(6.98,7.07]	(7.07,7.22]
	vegetation coverage index	0.0527	(0.17, 0.37]	(0.37, 0.51]	(0.51, 0.63]	(0.63, 0.74]	(0.74, 0.90]
	cultivation rate	0.0655	(0, 11]	(11, 33]	(33, 56]	(56, 80]	(80, 100]
	grazing capacity	0.0523	(0, 21]	(21, 59]	(59, 104]	(104, 155]	(155, 197]
Exposure (0.2055)	grassland area	0.0400	(0,0.11]	(0.11, 0.30]	(0.30, 0.53]	(0.53, 0.79]	(0.79, 1]
	farmland area	0.0540	(0,0.13]	(0.13,0.30]	(0.30, 0.59]	(0.59, 0.80]	(0.80,1]
	population density	0.0605	(0, 77]	(77, 222]	(222, 438]	(438, 1172]	(1172, 2844]
	economic density	0.0510	(0, 43]	(43, 110]	(110, 196]	(196, 340]	(340, 629]
Vulnerability (0.1992)	Soil physical and chemical properties of grassland	0.0571	(0.17, 0.24]	(0.24, 0.30]	(0.30, 0.38]	(0.38, 0.49]	(0.49, 0.67]
	Soil physical and chemical properties of farmland	0.0572	(0.18, 0.25]	(0.25, 0.32]	(0.32, 0.41]	(0.41, 0.51]	(0.51, 0.67]
	ratio of agricultural population	0.0448	(0, 86]	(86, 91]	(91,96]	(96, 98]	(98, 100]
	ratio of farming, forestry, husbandry and fishing outputs to GDP	0.0401	(0, 3]	(3, 8]	(8, 11]	(11, 14]	(14, 17]
Restorability (0.1865)	area of returning farmland to forests	0.0576	(0, 7.88]	(7.88, 12.00]	(12.00,13.83]	(13.83, 18.06]	(18.06, 29.14]
	population output	0.0339	(519, 4580]	(4580, 6090]	(6090, 6652]	(6652, 15412]	(15412, 19546]
	number of students	0.0434	(4519, 4602]	(4602, 4999]	(4999, 5745]	(5745, 6590]	(6590, 7650]
	ratio of sand-control inputs to GDP	0.0516	(0.68, 2.18]	(2.18, 2.77]	(2.77, 3.19]	(3.19, 4.25]	(4.25, 6.64]

A level zoning map of desertification disaster risk restorability in Naiman Banner is shown in Figure 5d. The area ratio of middle restorability is highest in the study area, it accounts for 36% of the total area. The second is very high restorability and high restorability, which accounts for 22% and 19% of the total area, respectively. Low restorability and very low restorability comprise 13% and 1% of the total area, respectively. Very high restorability area is mainly distributed in west-central and southwest part of the area. More students, higher population outputs and the sand-control inputs ratio resulted in this area having very high restorability. High restorability is mainly distributed in the northwest and the south of the study area. The area of returning farmland to forests and the population outputs are high in this area. Middle vulnerability is distributed with the “S” shape throughout the whole study area. The low and very low restorability is distributed in the east and the north part of the study area, respectively. The low and very low restorability in this area is due to a lower sand-control inputs ratio and the area of returning farmland to forests.

### 5.2. Assessment and zoning of Overall Desertification Disaster Risk

**Figure 6:**
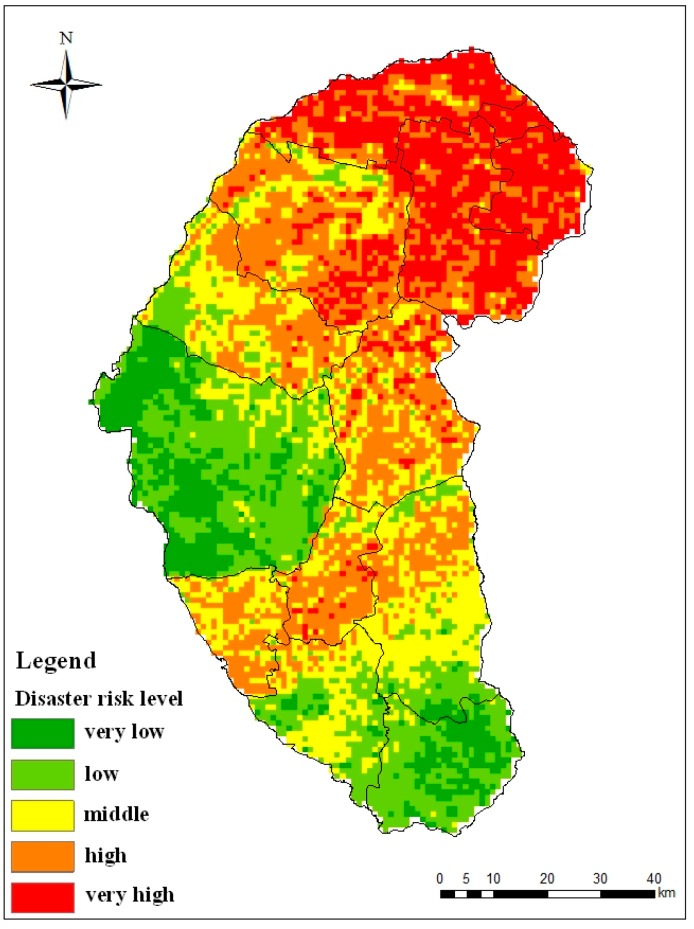
Zoning map of the desertification disaster risk in Naiman Banner.

**Figure 7:**
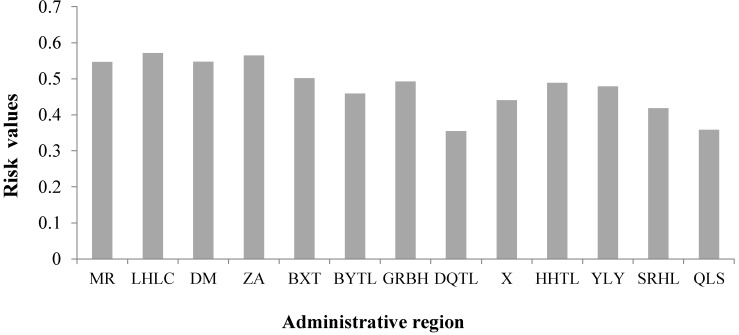
The desertification disaster risk in administrative region of Naiman Banner, which are minimum risk values of the Mingren (MR), Liuhaolinchang (LHLC), Dongming (DM), Zhian (ZA), Baxiantong (BXT), Bayintala (BYTL), Guribenhua(GRBH), Daqintala (DQTL), Xin (X), Huanghuatala (HHTL), Yilongyong (YLY), Sharihaolai (SRHL) and Qinglongshan (QLS).

Desertification disaster risk is a result of the concerted action of the four factors, so analysis and assessment of single factor is not enough for our research. Level zoning maps with the four factors were superimposed by their weights to compose the zoning map of desertification disaster risk. The map will provide a theoretical basis for policy makers and local desertification prevention and control work. We calculated the weights of the four factors by using the entropy combination weighted method. We then got the desertification disaster risk index of Naiman Banner by using Formula (15). We determined the risk grading standards of the disaster risk by using the optimal segmentation method (Table 3). The zoning map of desertification disaster risk is shown in Figure 6. In addition, we counted the ratio of different levels of risk area to the study area (Table 4) and mean value of the desertification disaster risk in each administrative region (Figure 7).

**Table 3:** Grading standards of the desertification disaster risk in Naiman Banner.

Level	Very Low	Low	Middle	High	Very High
Range	(0, 0.29]	(0.29, 0.47]	(0.47, 0.63]	(0.63, 0.78]	(0.78, 1]

The zoning map of the desertification disaster risk is shown in Figure 6. The area ratio of high risk is highest in the study area, it accounts for 28% of the total area. The middle risk area accounts for 23% of the study area while very high risk accounts for 18%. The low risk area and very low risk area comprise 21% and 10% of the total area, respectively. We can see from Figure 6 that the very high risk area is mainly distributed in the northeast of the study area and it is formed by the high hazard, vulnerability and low restorability. High and middle risk areas are distributed with the “S” shape in the study area and the risk here is influenced by high vulnerability, exposure and moderate restorability. The low and very low risk areas are mainly distributed in the west-central and southwest part of the study area and it is due to the low hazard, vulnerability, exposure and high restorability.

The mean value of the desertification disaster risk in administrative region of Naiman Banner is shown in Figure 7. Administrative regions of Mingren, Baxiantong, Dongming, Liuhaolinchang and Zhian located in the northern part of the study area and have a very high desertification disaster risk; Bayintala, Huanghuatala, Guribenhua and Yilongyong have a high desertification disaster risk; Sharihaolai and Xinzhen have a low desertification disaster risk; Daqintala and Qinglongshan have a very low desertification disaster risk. We can see that other parts of Naiman Bainner are prone to desertification disaster except the three towns in the southern part and the Daqintala.

**Figure 8:**
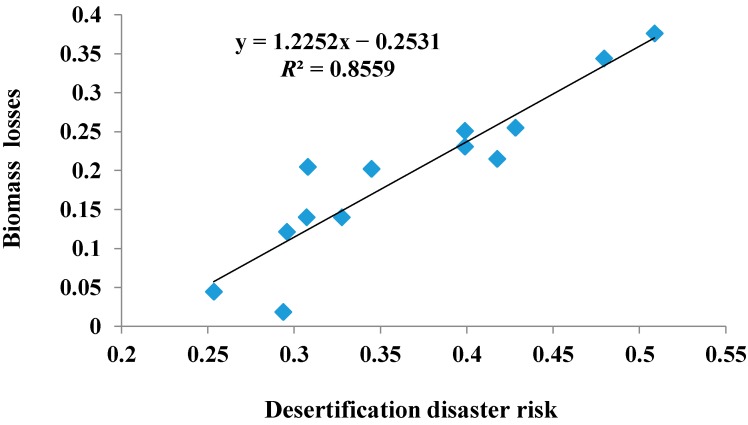
Comparing with the desertification disaster risk index and the biomass losses in Naiman Banner.

Precision validation for applicability of the desertification disaster risk assessment method and the model mentioned in this paper is necessary. Desertification disaster can lead to land degradation, biomass decreasing and the economic losses, *etc.* Considering the sensitivity and the availability of these indexes, we calculated the biomass losses caused by desertification disasters in the study area from 1980 to 2010, inspecting the result of the desertification disaster risk assessment. We selected TM images in year 1980 and 2010 to calculate biomass and find biomass losses in part by using two images, and then we calculated the average value of the biomass losses of the each administrative unit by using the partition statistics module of ArcGIS. Correlation analysis between the mean value of the desertification disaster risk index and the biomass losses is shown in Figure 8. As illustrated in Figure 8, a linear correlation existed between DDRI and the biomass losses (*R*^2^ = 0.8559, *p* < 0.01).We inferred that the biomass loss due to desertification disaster is similar to the desertification disaster risk. It is thus clear that the indicator system, the assessment model, the zoning map of hazard, exposure, vulnerability and the restorability calculated by fuzzy comprehensive evaluation method, and the zoning map of desertification disaster risk are is reasonable and feasible.

**Table 4:** The area ratio of the desertification disaster risk types in Naiman Banner.

Level	Very Low Risk Area	Low Risk Area	Middle Risk Area	High Rsk Area	Very High Risk Area
Ratio	10%	21%	23%	28%	18%

## 6. Conclusions

The main conclusions of this paper are as follows.

(1) Based on the principle of natural disaster risk and the desertification disaster formation mechanism, we have built the conceptual framework of desertification disaster risk. The desertification disaster risk was composed of four factors: hazard, exposure, vulnerability and restorability.

(2) We have built an indicator system of desertification disaster risk assessment with 19 indicators from the aspects of four risk factors. Then, we established the Desertification Disaster Risk Index (DDRI).

(3) We assessed the desertification disaster risk of Naiman Banner. The results showed that a high risk area is largest in the study area, and accounts for 28% of the total area, a middle risk area accounts for 23% of the total area and they are distributed with an “S” shape in the study area. The low risk area and very low risk area comprise 21% and 10% of the total area, mainly distributed in the west-central and southwest part. The very high risk area accounts for 18% of the study area and it is distributed in the northeast.

On the one hand, the author first combined the desertification disaster with natural disaster risk theory and assessed the desertification disaster risk. Different from the desertification degree assessment in the past, anticipation of desertification risk assessment has more significance for desertification prevention and control. On the other hand, this paper considered the natural process of desertification as well its disaster-causing ability and interaction between socioeconomic developments. This is the innovation of this paper. Furthermore, this paper realized multi-source data fusion. Remote sensing data, meteorological data, statistical data and experimental data are used in this paper. We identified that the theory and method used in this paper is feasible by using precision verification. However, only performed the correlation analysis between the administrative regions, and have not correlation analysis between the grids for the limitations of getting data. We need to consider this problem deeply in future research.
